# Development of a *Piscirickettsia salmonis* immersion challenge model to investigate the comparative susceptibility of three salmon species

**DOI:** 10.1111/jfd.13261

**Published:** 2020-10-16

**Authors:** Amy Long, Aidan Goodall, Simon R.M. Jones

**Affiliations:** ^1^ Fisheries and Ocean Canada Nanaimo BC Canada

**Keywords:** Atlantic salmon, immersion challenge, pink salmon, *Piscirickettsia salmonis*, sockeye salmon

## Abstract

*Piscirickettsia salmonis*, the aetiological agent of salmonid rickettsial septicaemia (SRS), is a global pathogen of wild and cultured marine salmonids. Here, we describe the development and application of a reproducible, standardized immersion challenge model to induce clinical SRS in juvenile pink (*Oncorhynchus gorbuscha*), Atlantic (*Salmo salar*) and sockeye salmon (*O. nerka*). Following a 1‐hr immersion in 10^5^ colony‐forming units/ml, cumulative mortality in Atlantic salmon was 63.2% while mortality in sockeye salmon was 10%. Prevalence and levels of the bacterium in kidney prior to onset of mortality were lower in sockeye compared with Atlantic or pink salmon. The timing and magnitude of bacterial shedding were estimated from water samples collected during the exposure trials. Shedding was estimated to be 82‐fold higher in Atlantic salmon as compared to sockeye salmon and peaked in the Atlantic salmon trial at 36 d post‐immersion. These data suggest sockeye salmon are less susceptible to *P. salmonis* than Atlantic or pink salmon. Finally, skin lesions were observed on infected fish during all trials, often in the absence of detectable infection in kidney. As a result, we hypothesize that skin is the primary point of entry for *P. salmonis* during the immersion challenge.

## 1 INTRODUCTION

*Piscirickettsia salmonis*, the aetiological agent of piscirickettsiosis, is a Gram‐negative, facultative intracellular bacterium that infects marine fish (Fryer, Lannan, Giovannoni, & Wood, 1992). Outbreaks of salmonid piscirickettsiosis, or salmonid rickettsial septicaemia (SRS), occur in farmed salmonids in Canada, Chile, Ireland, Norway and Scotland and tend to follow stressful environmental events such as algal blooms and elevated temperatures (Branson & Nieto Diaz‐Munoz, 1991; Cusack, Groman, & Jones, 2002; Rozas & Enríquez, 2014). SRS causes significant economic losses in the Chilean salmon farming industry (Rozas & Enríquez, 2014) whereas outbreaks in farmed salmonids elsewhere tend to be of lower severity. Susceptible salmonid species include pink salmon (*Oncorhynchus gorbuscha*), rainbow trout (*O. mykiss*), coho salmon (*O. kisutch*), chinook salmon (*O. tshawytscha*) and Atlantic salmon (*Salmo salar*).

In western Canada, *P. salmonis* has been reported in wild and cultured Pacific salmon including Chinook and pink salmon as well as in cultured Atlantic salmon (Brocklebank, Evelyn, Speare, & Armstrong, 1993; Brocklebank, Speare, Armstrong, & Evelyn, 1992). Between 2002 and 2016, there were a total of 36 farm‐level diagnoses of SRS in British Columbia (BC) (Jones, 2019). There have been no reports of SRS outbreaks in sockeye salmon (*O. nerka*), and the susceptibility of sockeye salmon to *P. salmonis* is unknown.

Controlled exposure studies have been used to understand transmission characteristics of *P. salmonis*, host–pathogen interactions and the evaluation of potential treatments (Rozas & Enríquez, 2014). The controlled laboratory infection is initiated in naïve fish by intraperitoneal (ip) injection or by cohabitation, in which recipient fish are held together with injected donors. However, ip injection bypasses primary host defences and typically elicits a rapid and severe infection, whereas mortality rates of cohabitation‐infected fish are variable, time to death is unpredictable, and the dose is difficult to quantify or standardize. In earlier attempts to develop a waterborne *P. salmonis* challenge model, the challenge organism was obtained from infected cell cultures and mortality rates were highly variable (Birkbeck, Rennie, Hunter, Laidler, & Wadsworth, 2004; Smith et al., 1997, 2015). The advent of enriched blood agar media for plate‐based culture of the bacterium (Mauel, Ware, & Smith, 2008) has provided an opportunity to obtain bacterial cells at concentrations sufficiently high for immersion challenges in combination with plate‐based methods for bacterial quantification.

The current study evaluates a reproducible waterborne *P. salmonis* challenge model in pink, sockeye and Atlantic salmon smolts. We document clinical symptoms and pathogenesis, report species‐specific differences in mortality and estimate the bacterial burden in tank water during the infections.

## 2 MATERIALS AND METHODS

### 2.1 Fish care

Fish were maintained in accordance with recommendations in the Canadian Council on Animal Care Guide to the Care and Use of Experimental Animals and approved by the Pacific Region Animal Care Committee (AUP 18‐021A1). All experimentation was conducted at the Pacific Biological Station (PBS; Nanaimo, BC). Separate exposure trials were conducted with pink salmon (Trial 1; April 2019), Atlantic salmon (Trial 2; June 2019) and sockeye salmon (Trial 3; September 2019). A fourth trial was conducted with Atlantic and sockeye salmon (Trial 4; December 2019). For each trial, water temperature, salinity, mean fish weight, stock density, bacterial concentration, number of fish per tank and tanks per dose are listed in Table 1.

**TABLE 1:** Salmon species, water quality and experimental parameters for individual trials

Trial	Species	No. of fish per tank	Mean temperature (°C)	Mean salinity (ppt)	Mean weight (g)	Stock density (kg/m^3^)	Dose (cfu/ml)	No. of tanks per dose
1	Pink	16	15.4	30.0	205	8.2	10^1^ 10^2^ 10^3^ 10^4^	2
2	Atlantic	25	15.0	30.0	163	10.2	10^5^	3
3	Sockeye	25	15.0	29.6	114	7.1	10^2^ 10^3^ 10^4^ 10^5^	2
4	Sockeye; Atlantic	15; 15	15.2	29.8	204; 261	3.1; 3.9	10^5^, 10^5^	1;1

Pink salmon fry were transferred to PBS from the Quinsam River Hatchery (BC) and held in seawater for approximately 12 months prior to Trial 1. Juvenile Atlantic salmon were obtained from a commercial hatchery on Vancouver Island and reared in brackish water at PBS. Four weeks prior to Trial 2, fish were transitioned to seawater. Sockeye salmon (Pitt River stock) were obtained from the Inch Creek Hatchery (BC), reared in 5°C dechlorinated freshwater for 12 months and transitioned to seawater 12 weeks prior to Trial 3. Atlantic and sockeye smolts previously transitioned to seawater were used in Trial 4.

During an individual trial, fish were held in 400‐L tanks provided with UV‐treated flow‐through seawater (7–8 L/min) under a 12‐hr:12‐hr photoperiod and fed a commercial diet (EWOS Canada) at daily rates of 1% (Trials 1 and 2) or 0.5% (Trials 3 and 4) total biomass. Dissolved oxygen levels were monitored daily and were ≥8 mg/L throughout the trial.

### 2.2 Bacterial culture

The SR‐1 isolate of *P. salmonis* used in all challenges was isolated in 2000 from Atlantic salmon during an SRS outbreak at a net‐pen aquaculture site near Vancouver Island. For all challenges, a low‐pass stock previously stored at −80°C was thawed and inoculated onto a monolayer of CHSE‐214 cells (ATCC CRL‐1681) (Lannan, Winton, & Fryer, 1984). Cells were grown in a 25‐cm^2^ flask with minimal essential medium (MEM) with Earle's salts (Gibco®) supplemented with 10% foetal bovine serum (MEM‐10; Gibco®) and 2.5 µg/ml Fungizone® (Amphotericin B; Gibco®). Flasks were incubated at 15°C for 15–17 d to allow for development of confluent cytopathic effect. The infected cells were then gently scraped off the bottom of the flask into the media using a cell scraper, and 100 µl of the harvested material was spread‐plated on BCG agar (Mauel et al., 2008). Plates were incubated at 15°C for 14–21 d to allow for development of a bacterial lawn.

### 2.3 Waterborne challenge

For all trials, *P. salmonis* colonies were resuspended in 105 ml of BM4 liquid medium (Henríquez et al., 2013) to an optical density (OD_600_) of 0.5. To prepare the immersion inoculum, 90 ml of resuspended bacterial cells was added to 810 ml of BM4. The OD_600_ measurement of this inoculum was ~0.05. The number of colony‐forming units/ml (cfu/ml) of the inoculum was determined by serially diluting in BM4 and spread plating 100 µl of each dilution onto BCG agar in duplicate. Plates were incubated at 15°C for 14–21 d and the number of colonies counted.

Prior to each trial, the water flow to each tank was stopped and 0.1 mg/L metomidate hydrochloride (Aquacalm; Syndel Canada) was added to the tank water as a sedative. After 15 min, an appropriate volume of the bacterial inoculum was added to the tank to obtain the desired final concentration. Supplemental aeration was provided throughout the challenge, and after 1 hr, water flow to the tanks was resumed. In Trials 1 and 3, there was a single negative control tank in which fish were immersed in 400 ml of sterile BM4 broth. There was no negative control tank in Trials 2 and 4.

In all trials, fish were monitored daily for mortality or symptoms of disease including uncoordinated swimming, lethargy, abnormal respiratory rate and lesions. Fish displaying visible symptoms of disease were killed by immersion in 400 mg/L tricaine methanesulphonate (MS 222; Syndel Canada). The number of dead and moribund fish removed daily contributed to the cumulative mortality and morbidity for each tank.

Dead, moribund and surviving fish were examined for evidence of infection and/or pathology. In Trial 2, fish were frozen until necropsy, and in all other trials, fresh fish were examined. At the conclusion of Trial 1, kidney samples were collected from 25% of fish (*n* = 8) in the 10^1^, 10^2^ and 10^3^ cfu/ml treatments and from all fish in the 10^4^ cfu/ml treatment (*n* = 32). In Trial 2, kidney and liver samples were collected and preserved in 95% ethanol from all survivors (*n* = 27) and from 34% of mortalities (*n* = 16), and kidney, liver and spleen from 5 mortalities were streaked on BCG agar. In Trial 3, kidney samples from all mortalities (*n* = 9) were preserved in 95% ethanol, and kidney, liver and spleen from 6 mortalities were streaked on BCG agar. In addition, subsamples of kidney from 20% of survivors in each treatment (*n* = 10) were streaked on BCG agar and preserved in 95% ethanol, respectively. At the conclusion of Trial 4, samples of kidney, spleen, liver, heart, brain, gill, intestine and skin lesions from each fish were preserved in 10% neutral‐buffered formalin (NBF) for histological examination. In addition, subsamples of kidney were streaked on BCG agar and preserved in 95% ethanol. BCG plates were incubated at 15°C for 14 – 21 d and monitored for bacterial growth.

### 2.4 Water sampling for bacteria

In Trials 2 and 3, water samples were collected every 2 d. To do so, the water flow to each tank was stopped for 30 min after which a 40 ml water sample was collected. Fish were monitored throughout this period, and supplemental aeration was provided. Water samples were kept at −80°C pending DNA extraction.

### 2.5 Histology

NBF‐fixed tissue samples were dehydrated in an alcohol gradient, clarified in xylene and infiltrated with paraffin wax for subsequent sectioning at 3 µm, staining and examination by light microscopy. Histological sections were stained with either haematoxylin and eosin, Giemsa or Gram stain (Jones, Long, MacWilliams, Polinski, & Garver, 2020). The presence and distribution of bacterial cells in histological sections were assessed by in situ hybridization (ISH) (Venegas, Contreras, Larenas, & Smith, 2004) with the following modifications. Post‐fixation of samples after proteinase K digestion was not done, and the labelled probes (Integrated DNA Technologies) and sheep antidigoxigenin alkaline phosphatase conjugate (Sigma‐Aldrich) were used at a final concentration of 1:500. The counterstain was 1% Light Green SF Yellowish (Sigma‐Aldrich).

### 2.6 DNA extraction and quantitative PCR

Thawed water samples were centrifuged at 12 000 x *g* for 10 min at 4°C, and the supernatant was removed. DNA was extracted from the resulting pellet and from cultured bacteria by resuspending in 180 µl Buffer ATL and 20 µl Proteinase K (Qiagen DNeasy® Blood and Tissue kit) following the manufacturer's instructions for Gram‐negative bacteria. DNA was purified from ethanol preserved kidney using the same kit and following the manufacturer's instructions for purification of total DNA from animal tissues. Eluted DNA was stored at −20°C.

Bacterial loads in tissue and tank water samples were determined using a qPCR assay designed for the *P. salmonis* 23S gene (Corbeil, McColl, St, & Crane, 2003). Individual reactions consisted of 1X TaqManTM Universal PCR Master Mix (Applied Biosystems), 900 nM each of the forward and reverse primer, 250 nM of the probe, 2 µl DNA template and nuclease‐free water for a final reaction volume of 25 µl. Reactions were run in duplicate on a StepOne‐Plus real‐time detection system (Applied Biosystems) following the manufacturer's protocol. For each qPCR run, the number of copies per reaction (c/rxn) was determined from a standard curve generated by amplifying a 10‐fold serially diluted double‐stranded DNA gBLOCK fragment (Integrated DNA Technologies), which included the *P. salmonis* primer and probe binding sites, ranging from 10^7^ to 10^1^ c/rxn. The limit of detection (LOD) for this assay is 5 c/rxn (Jones et al., 2020). Only samples with values at or above the LOD were considered positive. The bacterial burden, estimated by number of genome equivalents, was calculated by dividing c/rxn by six based on the reported replication of the 5S‐16S‐23S rRNA operon in the *P. salmonis* genome (Nourdin‐Galindo et al., 2017). Bacterial burden in tank water was normalized to sample volume and number of fish per tank.

The identity of cultured bacteria isolated from infected fish (1 sockeye and 1 Atlantic salmon) was confirmed by amplifying a fragment of the 16S ribosomal gene by conventional nested PCR (Mauel, Giovannoni, & Fryer, 1996) followed by sequencing (Eurofins Genomics). The resulting sequences were compared to NCBI nucleotide archives by BLAST.

### 2.7 Statistical analysis

For Trial 1 data, a chi‐square test of independence was done in R version 3.6.1 to determine the statistical significance of differences in the proportion of fish with lesions between treatments. For post hoc analysis, pairwise chi‐square tests using Fisher's exact test were employed and Bonferroni‐adjusted *P* values were generated using the rcompanion package (Mangiafico, 2020). Results were considered significant if *p* ≤ .05. Graphs were prepared in R using the ggplot2 package (Wickham, 2016).

## 3 RESULTS

### 3.1 Challenge trials

The nucleotide sequence of the 16S ribosomal gene from both bacterial isolates was identical to the corresponding region in the recently sequenced *P. salmonis* SR‐1 genome (GenBank accession number CP039227.1).

### 3.2 Trial 1 (pink salmon)

The trial was terminated at 30 d post‐immersion (dpi), and there were no mortalities. At this time, external lesions such as scale loss, areas of redness and ulcers were observed on fish in all groups with the exception of the negative control. Ulcers were typically shallow and bloody. Erosion of the dermal layer with exposure of the underlying muscle was rarely observed. Lesion frequency increased significantly (*χ* ^2^ = 66.2, *df* = 3, *p* < .001) with dose (Table 2). The number of fish with detectable *P. salmonis* also increased with dose (Table 2).

**TABLE 2:** *Piscirickettsia salmonis* in pink salmon (Trial 1). Number of fish with lesions, number positive in kidney by qPCR and median kidney bacterial loads at 30 days post‐immersion

Treatment	No. with lesions (No. examined)	No. positive by qPCR (No. examined)	Median GEq/µg DNA (IQR)
Negative control	0 (16)	0	0
10^1^	3 (32)^a^	0	0
10^2^	4 (32)^a^	1 (8)	1.5 x 10^3^
10^3^	21 (32)^b^	4 (8)	1.1 x 10^4^ (5.6 x 10^4^)
10^4^	30 (32)^b^	19 (32)	2.9 x 10^2^ (1.8 x 10^3^)

Note

Superscripts denote significant difference in proportion of lesions between treatments, *p* < .05.

### 3.3 Trial 2 (Atlantic salmon)

**FIGURE 1:**
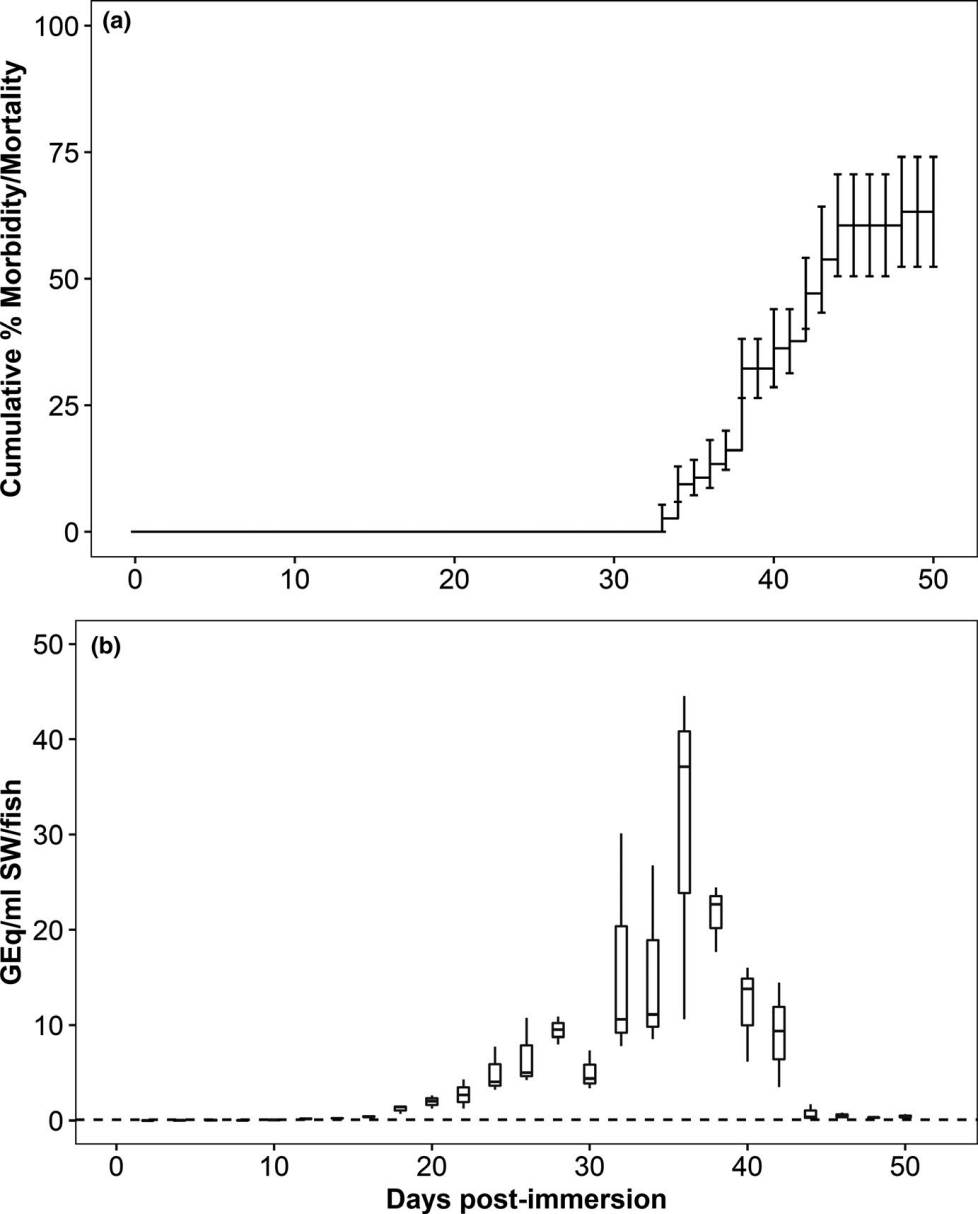
Mortality and bacterial burden in water following immersion of Atlantic salmon in *Piscirickettsia salmonis* (Trial 2). (a) Cumulative mean per cent morbidity and mortality; error bars denote standard error. (b) *P. salmonis* genome equivalents in tank water over the course of the trial. Data are presented in box plots in which the inner horizontal line is the median, and the upper and lower boundaries of the box correspond to the first and third quartiles. The upper and lower whiskers denote the largest and smallest values no further than 1.5 times the interquartile range. Dashed horizontal line denotes assay limit of detection

The trial was terminated at 50 dpi. Mean cumulative mortality and morbidity (CMM) were 63.2% ± 10.8 (Figure 1a), and mean days to death (MDD) was 39.6 dpi. The external lesions observed on all dead fish included scale loss, ulcers, darkening of skin around the ulcer, petechial haemorrhaging and areas of redness. Ulcer severity ranged from shallow, in which the dermal layer was still intact, to deep in which the underlying muscle was exposed. The bacterium was detected by qPCR in 33% of survivors sampled at 50 dpi and in all mortalities (Table 3). In addition, pure cultures of *P. salmonis* were re‐isolated on BCG agar from 5 mortalities sampled at 44 dpi.

**TABLE 3:** *Piscirickettsia salmonis* in Atlantic salmon (Trial 2; 10^5^ cfu/ml). Number of fish with lesions, number positive in kidney by qPCR and median kidney bacterial loads in dead and surviving salmon

Outcome	No. with lesions (No. examined)	No. positive by qPCR (No. examined)	Median GEq/µg DNA (IQR)
Mortalities	48 (48)	16 (16)	1.6 × 10^3^ (1.9 × 10^4^)
Survivors	23 (27)	9 (27)	8.0 (2.3 × 10^1^)

### 3.4 Trial 3 (sockeye salmon)

The trial was terminated at 58 dpi. Mean CMM values of 8% and 10% were observed in the 10^4^ and 10^5^ cfu/ml groups, respectively (Table 4), and MDD values were 37.8 (10^4^ cfu/ml) and 42.4 (10^5^ cfu/ml). *P. salmonis* was re‐isolated on BCG agar from all 6 dead salmon examined. Among the dead fish, white subcapsular hepatic lesions were observed in one without external symptoms of disease. Mottled livers (*n* = 2), splenomegaly (*n* = 1) and pale intestine (*n* = 3) were observed among the remaining 5 dead fish. Ulcer severity (*n* = 5) ranged from shallow and pale to deep with muscle tissue exposed, and 8 of 9 mortalities were positive by qPCR (Table 4). The median genome equivalents (GEq)/µg DNA in mortalities from the 10^4^ cfu/ml treatment was 8.9 × 10^4^ and 5.2 × 10^3^ for mortalities in the 10^5^ cfu/ml treatment. The bacterium was not isolated from survivors, and prevalence, as determined by qPCR, was low (Table 4).

**TABLE 4:** *Piscirickettsia salmonis* in sockeye salmon (Trial 3)

Treatment (CFU/ml)	CMM	MDD	No. *P. salmonis* re‐isolated (No. sampled)	No. with lesions (No. examined)	No. positive by qPCR (No. examined)	Median GEq/µg DNA (IQR)
Negative control	0	0	0 (5)	0 (25)	0 (5)	0
10^2^	0	0	0 (10)	2 (50)	0 (10)	0
10^3^	0	0	0 (10)	25 (50)	2 (10)	9.9 (2.7)
10^4^	8 ± 4	37.8	0 (10)	22 (50)	1 (10)	30.6
10^5^	10 ± 2	42.4	0 (10)	24 (50)	1 (10)	7.6

Note

Cumulative mortality and morbidity ± standard error (CMM), mean days to death (MDD), number of re‐isolations, number of fish with lesions, number positive in kidney by qPCR and median kidney bacterial loads in survivors.

### 3.5 Trial 4 (Atlantic and sockeye salmon)

The trial was terminated at 29 dpi. The CMM for Atlantic salmon and sockeye salmon was 13% and 0%, respectively. The trial was terminated one day after the 2 mortalities occurred, and all data were analysed together. *P. salmonis* was isolated on BCG agar from 8 of 15 Atlantic salmon and from 1 of 15 sockeye salmon (Table 5). Skin lesions of varying severity were observed on all salmon of both species. The lesions were typically bloody and shallow with an intact dermal layer. In Atlantic salmon, splenomegaly was noted in 4 fish and pale circular subcapsular hepatic lesions were observed in a single fish. Splenomegaly and/or mottled livers were observed in 4 sockeye salmon. The bacterium was detected by qPCR in more than twice as many (10 of 15) Atlantic salmon compared with sockeye salmon (4 of 15) (Table 5). The single Atlantic salmon with hepatic lesions had the highest bacterial load in kidney (1.2 × 10^5^ GEq/µg DNA). Of the 4 Atlantic salmon with splenomegaly, 3 were positive by qPCR. Of the 4 sockeye salmon with internal symptoms of SRS, 2 were positive by qPCR.

**TABLE 5:** *Piscirickettsia salmonis* in Atlantic and sockeye salmon (Trial 4; 10^5^ cfu/ml). Number of re‐isolations, number of fish with lesions, number positive in kidney by qPCR and median kidney bacterial loads at 29 days post‐immersion

Species	No. *P. salmonis* re‐isolated (No. sampled)	No. with lesions (No. examined)	No. positive by qPCR (No. examined)	Median GEq/ µg DNA (IQR)
Atlantic	8 (15)	15 (15)	10 (15)	2.7 (2.8)
Sockeye	1 (15)	15 (15)	4 (15)	1.4 (1.9)

**FIGURE 2:**
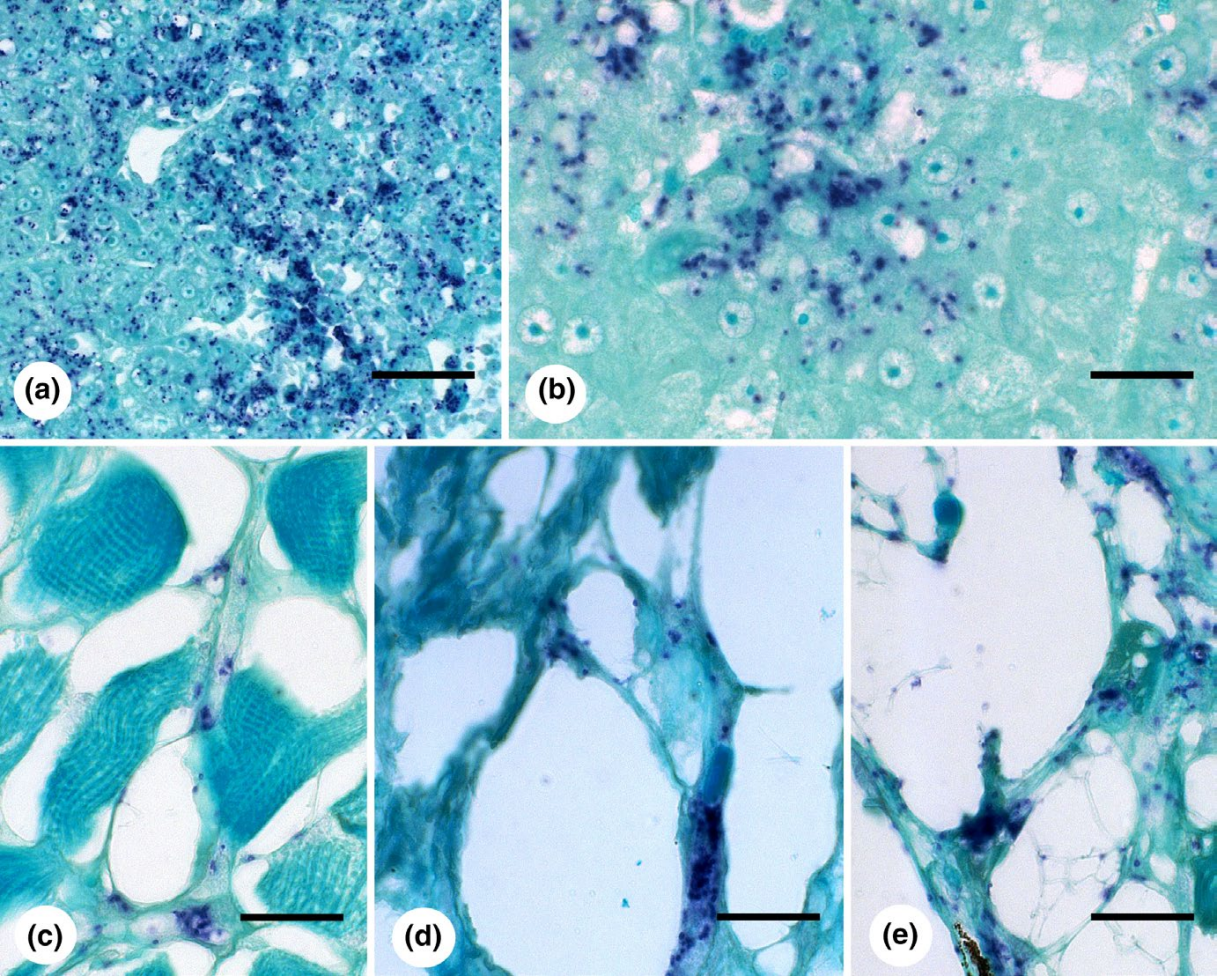
Histological sections from Atlantic salmon infected with *Piscirickettsia salmonis* visualized by in situ hybridization (Trial 4). (a) Low and (b) high magnification images of bacterial cells in liver. Bacterial cells associated with connective tissue of (c) skeletal muscle and (d, e) mesentery. Scale bars are 50 µm (a) and 20 µm (b‐d)

A histological preparation of liver from the most heavily infected Atlantic salmon revealed multiple zones of hepatocyte degeneration and necrosis. A section of this liver stained by ISH revealed a large number of bacterial cells that were free in necrotic lesions and intravacuolar within adjacent degenerate and intact hepatocytes (Figure 2a,b). In this salmon, small numbers of bacterial cells were observed intracellularly in sections of kidney and spleen and within cells associated with intestinal mesentery and similar loose connective tissue within skeletal muscle (Figure 2c‐e). In Giemsa‐stained sections, numerous bacterial cells were also observed in hepatocytes from this salmon in the absence of a lesion. Histological lesions were not evident in other tissues from this salmon or in histological liver sections from any other Atlantic salmon in which the kidney bacterial loads ranged from 3.3 × 10^0^ to 4.7 × 10^1^ GEq µg/DNA. Similarly, no lesions or bacterial cells were observed in histological liver sections of any sockeye salmon (kidney bacterial loads ranged from 0.5 to 2.1 GEq µg/DNA).

### 3.6 Quantification of P. salmonis in seawater

**FIGURE 3:**
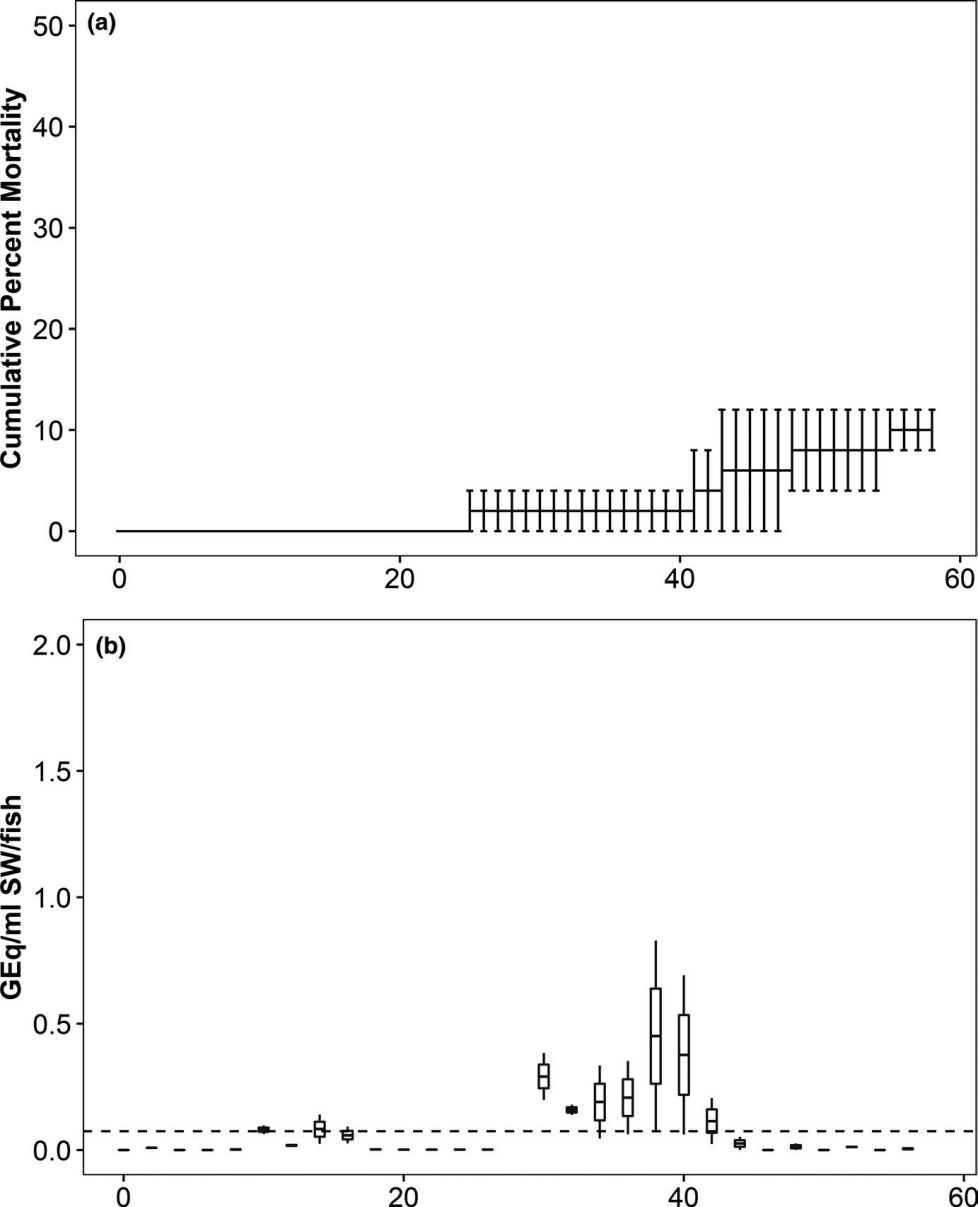
Bacterial burden in water following immersion of sockeye salmon in *Piscirickettsia salmonis* (Trial 3). *P. salmonis* genome equivalents in the (a) 1 × 10^5^ cfu/ml and (b) 1 × 10^4^ cfu/ml treatments. Data are presented in box plots in which the inner horizontal line is the median, and the upper and lower boundaries of the box correspond to the first and third quartiles. The upper and lower whiskers denote the largest and smallest values no further than 1.5 times the interquartile range. Dashed horizontal line denotes assay limit of detection

*Piscirickettsia salmonis* burden in tank water was estimated from samples collected during Trials 2 and 3. In Trial 2 (Atlantic salmon), the bacterial burden peaked at 36 dpi with a median value of 37.1 GEq/ml SW/fish (interquartile range, IQR = 16.9) (Figure 1b). In Trial 3 (sockeye salmon), the bacterial burden was consistently low in all treatments (Figure 3a,b). The bacterium was detected in the 10^5^ cfu/ml treatment between 30 and 42 dpi, with a peak of 0.5 GEq/ml SW/fish at 38 dpi (Figure 3a). In the 10^4^ cfu/ml treatment, *P. salmonis* was detected between 32 and 38 dpi and the bacterial burden peaked at 38 dpi at a median value of 0.19 GEq/ml SW/fish (Figure 3b). Fewer than 0.22 GEq/ml SW/fish were detected at 24 and 54 dpi in the 10^3^ treatment, and the bacterium was not detected in the 10^2^ treatment (data not shown).

## 4 DISCUSSION

In the current study, we demonstrate that 1‐hr immersion in a suspension of *P. salmonis* cells of western Canadian origin caused clinical SRS in pink, Atlantic, and sockeye salmon smolts and mortality in Atlantic and sockeye salmon smolts. Development of a standardized, reliable immersion challenge model for *P. salmonis* permits elucidation of pathogen transmission dynamics and evaluation of potential disease treatments. By demonstrating the virulence and infectivity of known concentrations of *P. salmonis* cultured on BCG agar and re‐isolating the organism from diseased fish, we confirmed the quantitative utility of this method. Previous efforts to develop immersion challenge methods for *P. salmonis* used inocula derived from cell culture and yielded variable results. No mortality was reported in 15 g rainbow trout (intact or scarified skin) following a 3‐min immersion in 5.7 *Piscirickettsia* 10^4^ median tissue culture infective doses (TCID_50_)/ml of a Chilean (LF‐89) strain (Smith et al., 1997). Despite the lack of mortality, infections were acquired and the authors suggested longer exposure times or higher bacterial concentrations may have been necessary to elicit mortality. A later study with 2.5 g rainbow trout reported 92% mortality following immersion in 10^5^ TCID_50_/ml of another Chilean strain (SLGO‐95) (Smith et al., 2015). In a study with 100 g Atlantic salmon post‐smolts, 1 of 10 fish died following 1‐hr immersion in 10^5^ TCID_50_/ml of a Scottish *P. salmonis* isolate (SCO‐95A) (Birkbeck et al., 2004). These observations indicate the importance of fish size and bacterial strain as considerations in immersion challenge development. This earlier work also highlights the limitations in calculating absolute bacterial infectious doses from cytopathic effect in cultured cells.

Our research has begun to shed light on the relative susceptibility of sockeye salmon to *P. salmonis* infection and SRS. Following exposure to 10^5^ cfu/ml, mortality in sockeye salmon (Trial 3) was 10% compared with 63% in Atlantic salmon (Trial 2). Similarly, at this dose the bacterium was detected in fewer sockeye survivors (10%, Trial 3) compared with Atlantic salmon survivors (33%, Trial 2). In Trial 4 in which sockeye and Atlantic salmon were concurrently challenged with 10^5^ cfu/ml, despite similarities in skin lesion frequency, bacterial isolation rates (7% versus 53%) and qPCR prevalence (27% versus 67%) were lower in sockeye salmon. Data from pink salmon exposed to 10^4^ cfu/ml in Trial 1 (qPCR prevalence = 59%, median GEq/µg DNA = 290) suggest higher susceptibility in this species. Together, these data are consistent with the hypothesis that sockeye salmon are less susceptible to *P. salmonis* than pink or Atlantic salmon and represent the first evidence of species‐specific differences in salmonid susceptibility to this pathogen when exposed via immersion. In an earlier study (Smith et al., 1996), size‐matched coho salmon and rainbow trout were ip‐injected with *P. salmonis* LF‐89 and susceptibility compared. Lower levels of cumulative mortality and reduced bacterial burden in kidney led to the conclusion that rainbow trout were less susceptible than coho salmon. In another study (Garcés et al., 1991), mortalities of up to 100% in coho and Atlantic salmon following ip injection with high doses (> 10^3.3^ TCID_50_/fish) of an unidentified Chilean *P. salmonis* isolate precluded any distinction of susceptibility between these species. In western Canada, outbreaks of SRS or a similar disease have been reported in cultured Atlantic salmon, Chinook salmon and coho salmon (Brocklebank et al., 1992), and the infection has been recognized in pink salmon (Brocklebank et al., 1992; Jones et al., 2020). In contrast, there have been no reports of SRS or *P. salmonis* infection in sockeye salmon and our data suggest a reduced likelihood of infection or SRS in migratory sockeye salmon.

Pathogen shedding data provide useful indications of temporal shifts in infectiousness within a population. In the current study, we estimated the timing and magnitude of *P. salmonis* shedding from water samples. In tanks containing infected Atlantic salmon, the bacterium was first detected at 12 dpi and levels peaked at 36 dpi, 3 d prior to the MDD. From 44 dpi onwards, levels of *P. salmonis* in the water column remained close to the LOD and coincided with the cessation of acute mortality. Similarly, peak shedding in sockeye salmon occurred at 38 dpi, 4 d prior to the MDD. The peak shedding rate in Atlantic salmon was 82‐fold higher than the peak shedding rate in sockeye salmon exposed to 10^5^ cfu/ml. We can therefore conclude that, in infected fish, the highest levels of *P. salmonis* are shed shortly before death, and Atlantic salmon experiencing an SRS outbreak are most infectious between 18 and 42 dpi. Our estimates of bacterial shedding in this study reflect the accumulation of bacteria in tank water from all infected fish. Shedding is unlikely to be synchronized either in timing or magnitude within a population and further studies are required to estimate the contributions of individual fish to better define infectious characteristics within the population. There are few data describing shedding of bacterial pathogens from fish. In *Renibacterium salmoninarum*, another facultative intracellular bacterium, McKibben and Pascho (1999) reported shedding from chinook salmon first occurred at 12 dpi and consistently occurred by 20 dpi.

Skin has previously been identified as one of the primary routes of entry of *P. salmonis* (Smith et al., 1999). The skin lesions described here were similar to those described in previous studies in which fish were exposed by skin patch, injection, oral inoculation or gill inoculation (Almendras, Fuentealba, Markham, & Speare, 2000; Smith et al., 1999). Skin lesions due to *P. salmonis* progressed from a slight raised area to decolouration and scale loss and finally to ulceration (Smith et al., 1999). When examined at or before the onset of mortality, more pink, sockeye and Atlantic salmon (Trials 1 and 4) had skin lesions than had detectable infections in the kidney. Almendras et al. (2000) had previously hypothesized that *P. salmonis* infects leucocytes and is disseminated throughout the circulatory system whereupon it infects vascular endothelial cells, eventually resulting in systemic infection. Leucocyte proliferation has been observed at the site of *P. salmonis*‐induced skin lesions (Smith et al., 1999), and *P. salmonis* survives and replicates in leucocytes (McCarthy et al., 2008; Rojas, Galanti, Bols, & Marshall, 2009). This information, combined with premortality shedding measured in Trials 2 and 3, is consistent with the delayed onset of a systemic infection after an infection is acquired by external exposure. Although we did not monitor the route of entry of the bacterium, our data are consistent with the hypothesis that skin serves as a portal of entry for the bacterium during the immersion exposure. The epithelia of gill, the oral cavity and the oesophagus have also been suggested as potential routes of entry (Almendras, Fuentealba, Jones, Markham, & Spangler, 1997; Rozas‐Serri et al., 2017; Smith et al., 1999), and further work is needed to elucidate their relative importance in uptake of the bacterium.

In conclusion, we describe the development and application of a standardized, reliable and controlled quantitative immersion challenge method, using a western Canadian isolate of *P. salmonis*, which results in infection and SRS in Atlantic salmon and two species of Pacific salmon. We link the timing and magnitude of bacterial shedding with acute mortality and provide evidence that sockeye salmon have reduced susceptibility to the infection and to SRS compared with Atlantic and pink salmon.

## CONFLICT OF INTEREST

The authors declare they have no conflict of interest.

## Data Availability

The data that support the findings of this study are available from the corresponding author upon reasonable request.
